# Using the Avocado as an Evening Snack to Investigate Whole Food Matrix and Macronutrient Composition on Morning Metabolic Indices in Adults With Prediabetes

**DOI:** 10.1016/j.cdnut.2025.107486

**Published:** 2025-06-12

**Authors:** Chelsea Preiss, Olga Marquis, Indika Edirisinghe, Britt M Burton-Freeman

**Affiliations:** Department of Food Science and Nutrition and Center for Nutrition Research, Illinois Institute of Technology, Chicago, IL, United States

**Keywords:** prediabetes, avocado, snack, triglycerides, postprandial, insulin, glucose

## Abstract

**Background:**

Evening snacks are consumed by most people. Prior research suggests that macronutrient composition influences next morning metabolic responses and beneficial effects are imparted by low glycemic index foods and/or those that contain fermentable fibers. Avocados are a complex matrix of healthy fats and fibers with low glycemic value. Prediabetes is an early stage of impaired glucose and insulin responses, and a critical period for dietary strategies to improve metabolic health.

**Objectives:**

This study aimed to assess evening snack macronutrient composition and whole food (avocado) matrix on morning metabolic indices in adults with prediabetes.

**Methods:**

Participants (*n* = 55; age: 44 ± 14 years; BMI: 28 ± 6 kg/m^2^) were randomly assigned to 1 of the 3 energy-matched (280 kcal) snack interventions in a crossover design: control (low fat, low fiber); avocado, whole (high fat, high fiber); and matrix control (high-fat, high-fiber combined ingredients). Snacks were consumed on 3 separate evenings at the same time (±1 h), followed by 12-h fast and blood collections before and after (3 h) a standard breakfast (720 kcal). Data were analyzed by repeated-measure analysis of variance using the mixed procedure.

**Results:**

Fasting and postprandial glycemic and inflammatory markers were not different after snack interventions (*P* > 0.05). After the avocado snack, fasting triglycerides tended to be lower (*P* = 0.09), and a snack-by-time interaction (*P* = 0.02) revealed significantly lower triglyceride concentrations at 3 h.

**Conclusions:**

Snacking on avocados in the evening may have important effects on triglyceride metabolism.

This trial was registered at clinicaltrials.gov as NCT05263011.

## Introduction

Over 60% of people who eat dinner report eating an after-dinner snack [1]. The timing of snacking has been investigated for effects on metabolic outcomes like glycemic and lipid responses. Some studies have shown that evening dietary interventions affect glucose regulation the following morning [[2], [3], [4], [5]]. Beneficial effects are typically shown when the meals and snacks have a low glycemic index (GI) value and/or contain fermentable fibers. A low-GI dietary intervention has been shown to reduce glycemic responses in meals the next day in young adult males [6]. Feeding times of meals and size of snacks are other variables being investigated on next-day glycemic and lipemic responses [7,8].

Avocados have close to no-GI value and are a rich source of healthy fats and fibers [9]. Avocados contain ∼9.2 g dietary fiber per 1 Hass avocado fruit (136 g), thus making it an easy addition to the diet to meet the recommended 14 g/1000 kcal/d [10]. Approximately 98 million Americans have prediabetes, (i.e. ∼1 in 3 adults), which is a major risk factor for type 2 diabetes [11]. With the prevalence of snacking behavior and conversion rates from prediabetes to type 2 diabetes, it is prudent to have a better understanding of physiologic responses to snacking in people in general, but particularly in people with prediabetes.

Fasting hyperglycemia or impaired fasting glucose refers to an elevated blood glucose concentration between 100 and 125 mg/dL after a prolonged period, such as an overnight fast, and is a defining trait of prediabetes [12,13]. Insulin resistance is a common underlying characteristic of prediabetes and a risk factor in the progression to type 2 diabetes, leading to elevated fasting blood glucose concentrations above 125 mg/dL [14]. Ingesting a snack before bedtime (evening snack) is proposed to reduce the fasting window and decrease the gluconeogenic demands on the liver [15]; however, this effect may be dependent on the composition of the evening snack. Consuming low-GI, high-fat, and high-fiber foods may extend insulin secretion overnight, supporting glycemic control the following morning and potentially throughout the day.

Our objective was to assess macronutrient composition and whole food (avocado) matrix of evening snacks on morning metabolic indices in adults with prediabetes. We hypothesized that consuming avocado as an evening snack will reduce peak glucose and area under the glucose concentration curve after a standardized breakfast meal compared with a calorie-equivalent, low-fat, low-fiber control snack. A secondary hypothesis tested the importance of the whole (food) unprocessed matrix of the avocado, investigating whole avocado compared with a macronutrient-matched ingredient-based snack on glycemic control.

## Methods

### Ethics, participants, and study design

The study was conducted following the principles set forth by the Declaration of Helsinki and approved protocols and consent forms by the institutional review board (IRB) of the Illinois Institute of Technology (protocol IRB_2021_81; approval date 21 March, 2021). Study procedures were initiated after the IRB-approved informed consent document was presented and signed by participants. All procedures were performed at the Clinical Nutrition Research Center (CNRC), Illinois Institute of Technology, Chicago, IL. The study was registered at clinicaltrials.gov (NCT05263011).

**FIGURE 1:**
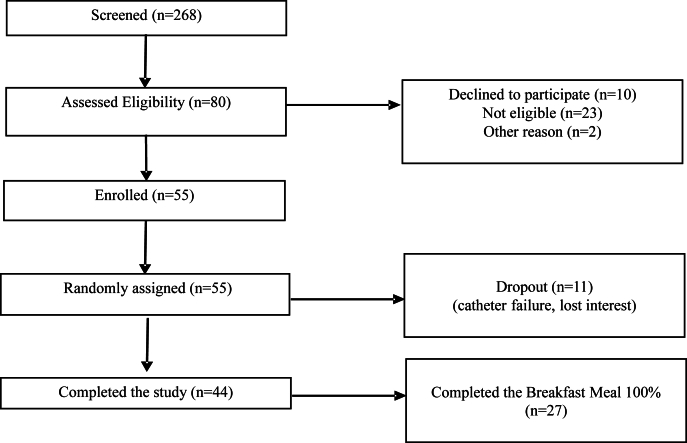
CONSORT diagrams for study recruitment.

Potential participants were screened for inclusion criteria: impaired fasting blood glucose defined by capillary or venous values between 100 and 125 mg/dL, age 25–70 years, and no clinical evidence of cardiovascular or metabolic disease. Individuals taking lipid-lowering medications, anti-inflammatory drugs, or dietary supplements that may interfere with the study outcomes or practice extreme dietary regimens and/or physical activity were deemed not eligible for the study. Figure 1 shows the number of participants who were recruited from the greater Chicagoland area from June 2022 to March 2023.

This was a randomly assigned, 3-arm crossover study. Each participant was randomly assigned to a sequence of 3 evening snack interventions: control snack (low fiber, low fat) consisting of low-fat Yoplait yogurt, vanilla (170 g), and Good Thins Simply Salt Rice Snacks Gluten Free Crackers (18 crackers); whole avocado (168 g; high fat, high fiber); and matrix control snack (high-fat, high-fiber) consisting of low-fat Yoplait yogurt, vanilla, (85 g), Good Thins Simply Salt Rice Snacks Gluten Free Crackers (1 cracker), Olive Oil, Extra Light Tasting (23.3 g), microcrystalline cellulose (7.84 g), and pectin (3.4 g)—on 3 different occasions using computer-generated randomly assigned sequences. Details about the 3 different interventions, including nutritional composition, are given in Table 1. Participants came to the CNRC for 6 visits, which were grouped as three 2-day visits corresponding to their randomization schedule. On the first day of the 2 days, participants picked up a dinner meal and/or the assigned evening snack intervention. Participants could re-create their dinner meal each time if they did not want a dinner provided. On the second day, which was the next morning, participants arrived fasted to participate in the postprandial multiblood sampling procedure. These 2-day visits occurred 3 times, ≥3 days apart but within 3 wk. Each postprandial study visit day lasted ∼3–4 h. Participants consumed only the standardized or their own approved dinner meal and the evening snack (intervention) provided by CNRC on the evening before the visit. The snacks were to be eaten by themselves with no other food added, and participants were instructed to eat the whole avocado plain and on its own. Participants were instructed to eat their randomly assigned evening snack ∼12 h before their visit day and their standardized dinner meal 14 h before their visit day. If a person had a visit day scheduled at 08:00, they would consume their evening snack at 20:00 and their standardized dinner meal at 18:00. Reminders of meal and snack timing were given out on the day of pickup. Meal and snack timing was considered within compliance if it was ±1 h of scheduled time, as assessed by researchers from a 1-d food diary the participant filled out the day before visit days. After the snack, participants were instructed to not eat or drink anything except plain water and to have a usual night of sleep, aiming for ≥7 h of sleep before coming in the next morning. Participants were also instructed to refrain from consuming caffeinated beverages, food items interfering with the study outcomes, maintaining the same exercise pattern, and avoiding alcoholic drinks 24 h before the postprandial study visit.

**TABLE 1:** Recipe and nutrient composition of evening snack interventions^1^.

	Avocado,whole^2^	Controlsnack^3^	Matrixcontrol^4^
Total carbohydrate (g)	14.6	57	16.9
Total sugars (g)	0	22	11
Total protein (g)	3.4	7	2.6
Total fat (g)	25.8	2.5	23.9
Saturated fat (g)	3.4	0.5	3.6
Polyunsaturated fat (g)	3.4	0	2.5
Monounsaturated fat (g)	16.8	0	16.6
Dietary fiber (g)	11.2	0	11.2
70% insoluble (calculate-g)	7.8	0	7.8
30% soluble (calculate-g)	3.4	0	3.4
Total energy (kcal)	280	280	282

1 Nutrients of food ingredients analyzed by Food Processor Pro SQL Edition by ESHA (version 10.15.41; ESHA Research).

2 Avocado (168 g).

3 Yogurt, Yoplait, low-fat, original, vanilla (170 g); Good Thins Simply Salt Rice Snacks Gluten Free Crackers (18 crackers).

4 Yogurt, Yoplait, low-fat, original, vanilla (85 g); Good Thins Simply Salt Rice Snacks Gluten Free Crackers (1 cracker); oil, olive, extra light tasting (23.3 g); microcrystalline cellulose (7.84 g); pectin (3.4 g).

### Postprandial study day visit

**FIGURE 2:**
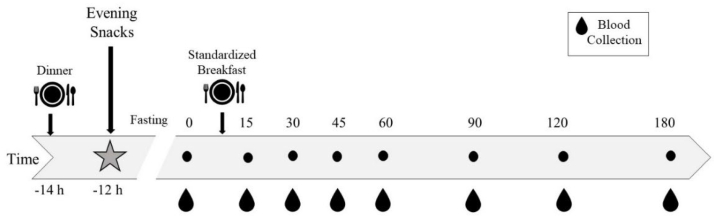
Study schema: randomized crossover design. Snacks provided the night before the blood collection study day.

At each postprandial study day visit, participants arrived at the CNRC fasted overnight after eating the evening snack intervention. After confirming compliance with study instructions, body weight, waist circumference, fasting finger prick for blood sugar, and vital signs were measured. Thereafter, an indwelling catheter was placed in the antecubital vein by a licensed health care professional, and a fasting blood sample was collected into EDTA vacutainers. After completing baseline blood sampling, participants were provided with a standard breakfast meal and instructed to finish within 10 minutes. Additional blood samples were collected at 15, 30, 45, 60, 90, and 120 min (Figure 2) after the initiation of the breakfast meal. All blood samples were collected through the intravenous catheter into EDTA tubes, placed on ice immediately, and centrifuged at 4 °C and 453 × *g* for 10 min within 30 min after the sample collection. Plasma was separated and stored at −80 °C until analysis. The recipe and major nutrient composition of the breakfast meal are given in Table 2.

**TABLE 2:** Recipe and nutrient composition of the breakfast meal^1^.

Ingredients or nutrients	Amount
Ingredients (g)	
Biscuits, buttermilk, cooked^2^	130
Butter, unsalted, sweet cream^3^	8
Apple jelly^4^	46
Egg whites	70
Cooking spray, original^5^	1
Cheddar cheese, sharp, finely shredded^6^	5
Apple juice^7^	237
Total energy (kcal)	720
Fat (%)	33
Carbohydrate (%)	59
Protein (%)	10

1 Nutrients of food ingredients analyzed by Food Processor Pro SQL Edition by ESHA (version 10.15.41; ESHA Research).

2 Pillsbury; General Mills.

3 Land O’Lakes; Land O’Lakes.

4 Smuckers; JM Smucker.

5 Pam; Conagra Brands.

6 Kraft; Kraft Foods.

7 Private Label; Albertsons Companies.

### Metabolic and inflammatory markers analysis

Glucose, insulin, and triglyceride (TG) were analyzed by Randox automated clinical analyzer following manufacturer’s instruction and using appropriate quality controls. Inflammatory markers (IL-6 and TNF-α) were measured using Luminex xMAP INTELLIFLEX high-sensitivity assays technology following manufacturer’s instructions and appropriate quality control samples.

### Statistical analysis

Statistical analyses, randomization schedules, and sample size estimates were performed using SAS, version 9.4 (SAS Institute). Demographic and baseline health indices were tabulated from descriptive statistics. Shapiro–Wilks tests and Probability-Probability (PP) and Quantile-Quantile (QQ) plots were used to assess the normality distributions of all outcome variables. Outcome variables that were not normally distributed were log_10_ transformed and retested before statistical analyses. Outliers were determined using a box plot review from the normality test and removed if >1.5 in the IQR. Prebreakfast blood samples from evaluable participants (*n* = 44) were tested for effects of the intervention on fasting glucose, insulin, TG, IL-6, and TNF-α using SAS PROC MIXED analysis of variance with intervention as the fixed factor and participant as the random variable. Covariates were tested and included in the final models if significant [e.g. age, BMI (in kg/m^2^), and sequence). Postprandial metabolic (glucose, insulin, peak glucose, area under the glucose curve, and TG) and inflammatory markers (IL-6 and TNF-α) were analyzed from blood samples collected before and after the breakfast meal using repeated-measure analysis of variance (RM-ANOVA), with intervention and time as the main factors. The evaluable data set for this analysis was 27 due to some participants not consuming the breakfast meal in its entirety. Covariates were tested and included as appropriate in the final models and noted in table footnotes. Results are presented as least square means ±SEMs unless indicated otherwise. Statistical significance was based on a 2-sided comparison of interventions at the 5% significance level under a null hypothesis of no difference between interventions. Multiple comparisons were corrected using Tukey–Kramer in mixed model procedures. Statistical significance was determined at *P* < 0.05.

Sample size was estimated from previous work assessing the acute postprandial effects of whole food (orange) compared with processed or ingredient components on peak glucose and incremental area under the curve (iAUC), which controls baseline variance by subtracting each person’s own baseline value from each time series value before calculating AUC [16,17]. To detect 8% difference in means of peak glucose or iAUC at α = 0.05 (corrected for multiple comparisons) and usual attrition, we estimated 42 people would be required for the study. Post hoc sample size analysis using Proc Power (SAS) for repeated-measure analyses and Cohen d for effect size independent of sample size were used to assess meaningfulness of statistical results in the reduced sample (*n* = 27) when appropriate.

## Results

### Participant demographics and characteristics

A total of 80 participants were assessed for eligibility, of which 55 met the inclusion criteria and were randomly assigned to a sequence order snacks would be provided. Eleven participants dropped out at 1 of the 3 evening snack interventions for various reasons, and 44 ate all 3 interventions. Of 44 participants, 27 participants ate the entire standardized breakfast meal at all 3 study day visits in its entirety (Figure 1). Demographic information and other health indices are given in Table 3.

**TABLE 3:** Baseline participant characteristics.

Characteristics	Total (*N* = 55)
Age (y)^1^	44 ± 15
Sex (*n*)	
Female	23
Male	32
Race (*n*)	
Asian	18
Hispanic/Latino	1
Caucasian	18
African American	12
Other	6
BMI^1^	28 ± 6
Underweight, <18.5 (*n*)	0
Normal, 18.5–24.9 (*n*)	20
Overweight, 25.0–29.9 (*n*)	15
Obese, ≥30 (*n*)	20
Waist circumference,^1^ midpoint (cm)	98 ± 15
Systolic blood pressure^1^ (mm Hg)	120 ± 15
Diastolic blood pressure^1^ (mm Hg)	74 ± 9
Fasting blood glucose,^1^ capillary (mg/dL)	101 ± 9

1 Values are mean ± SD.

### Fasting metabolic and inflammatory marker assessment the morning after consuming 3 different evening snack interventions

Fasting glucose, insulin, and inflammatory markers were not significantly different between the 3 evening snack interventions. However, a moderate trend for lower fasting TG was observed (*P* = 0.09), but was not significantly different between interventions (Table 4, *n* = 44).

**TABLE 4:** Fasting metabolic and inflammatory markers assessments in the morning after evening snack.

Fasting^1^ (before test meal) variable	Avocado, whole (*n* = 44)	Control (*n* = 44)	Matrix control (*n* = 44)	*P*-intervention
Glucose (mg/dL)	106.3 ± 1.2	105.3 ± 1.3	104.7 ± 1.2	0.47
Insulin (μIU/mL)	10.6 ± 0.9	10.7 ± 0.9	10.6 ± 0.9	0.34
Triglyceride (mg/dL)	83.3 ± 5.9	90.1 ± 5.9	77.5 ± 5.9	0.09
IL-6 (pg/mL)	1.74 ± 1.10	1.74 ± 1.10	1.86 ± 1.10	0.27
TNF-α (pg/mL)^2^	0.71 ± 1.04	0.68 ± 1.03	0.74 ± 1.03	0.17

1 Values are means ± SE.

2 BMI as a covariate.

### Postprandial metabolic assessments after a standardized breakfast meal the morning after consuming 3 different evening snack interventions

**FIGURE 3:**
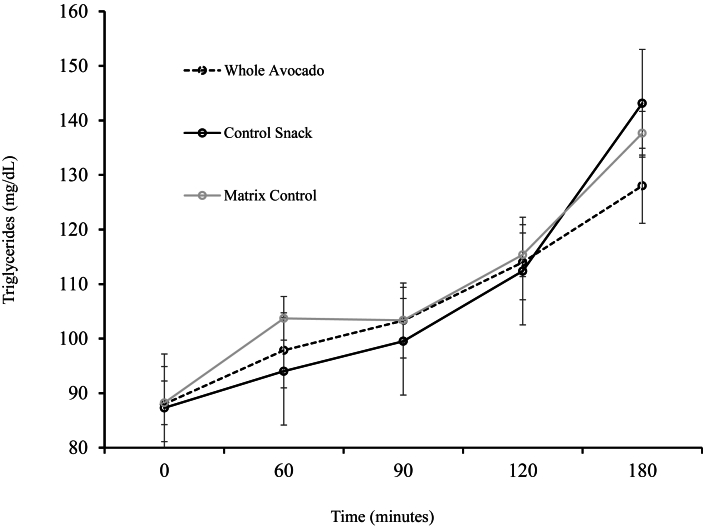
Postprandial triglyceride concentrations after a standard breakfast meal the morning after an evening snack: whole avocado, control snack, or matrix control snack, *n* = 27. Intervention × time interaction, *P* = 0.024. Test of effect slices at timepoint 180 min (*P* = 0.04). Avocado vs control snack: effect size: −15.1 ± 5.9 mg/dL; Cohen d: 0.70.

The effect of different evening snack interventions on standardized breakfast meal–induced postprandial glycemic and TG responses was assessed using RM-ANOVA methods as described earlier. Owing to participant variance in eating the entire breakfast meal, data are shown for only those participants who ate the breakfast meal at all 3 study visits in entirety (*n* = 27, Table 5). No significant effect of the different evening snack interventions was observed for any postprandial GIs [glucose, insulin, peak glucose, iAUC_(0–180)_ glucose; *P* > 0.05]. The postprandial TG response of those who ate the entire breakfast meal (*n* = 27) after an evening snack significantly differed between treatments related to time (intervention × time interaction, *P* = 0.02). Mean postprandial TG response values were 109.7 ± 3.2 mg/dL for matrix control snack; 107.3 ± 3.2 mg/dL, control snack; and 106.2 ± 3.2 mg/dL, avocado snack. The time × concentration curve for TG is shown in Figure 3. The test of effect slices indicated a significant difference between snacks at time point 180 min (*P* = 0.04). Cohen d analysis indicated that the TG concentration effect size at 180 min between the avocado and control snack interventions was 0.70, a moderate to large effect size. The power to detect an effect was 79% for this outcome with sample size of *n* = 27.

**TABLE 5:** Postprandial metabolic and inflammatory marker responses to a breakfast meal the morning after evening snack.

Postprandial^1^ (after test meal) variable	Avocado, whole (*n* = 27)	Control (*n* = 27)	Matrix control (*n* = 27)	*P* (treatment × time)
Glucose (mg/dL)	126.6 ± 2.9	125.3 ± 2.9	128.0 ± 3.0	0.99
Insulin (μIU/mL)^2^	47.4 ± 1.7	48.2 ± 1.7	47.9 ± 1.6	0.98
Triglyceride (mg/dL)	106.2 ± 3.2	107.3 ± 3.2	109.7 ± 3.2	0.02
IL-6 (pg/mL)	1.8 ± 1.1	2.0 ± 1.1	1.8 ± 1.1	0.68
TNF-α (pg/mL)^3^	1.84 ± 1.1	1.1 ± 1.1	1.85 ± 1.1	0.68
Peak glucose (mg/dL)	167.8 ± 4.7	164.8 ± 4.7	168.6 ± 4.7	0.56
iAUC glucose (mg.h/dL)	56.4 ± 10.1	49.8 ± 10.1	56.4 ± 10.1	0.93

1 Values are means ± SE of all time point values (0–180 min) except peak glucose and iAUC_(0–180)_ glucose, which are means ± SE of single point or calculated values, respectively.

2 Race is a covariate.

3 BMI is a covariate.

### Inflammatory markers assessments in response to a standardized breakfast meal after 3 different evening snack interventions

The effect of different evening snack interventions on a standardized breakfast meal–induced postprandial inflammation was assessed using RM-ANOVA methods as described earlier. Data are given in Table 5. Data were analyzed for those who ate the entire breakfast after an evening snack (*n* = 27). No significant effect of the interventions was observed for any inflammation variables (IL-6 and TNF-α; *P* > 0.05).

## Discussion

We hypothesized that consuming avocado, a whole unprocessed food with high-fiber, high-fat content, as an evening snack would reduce the glucose excursion to the first morning meal compared with a calorie-equivalent, low-fat, high-carbohydrate, low-fiber control. A secondary hypothesis was that the matrix of the whole fruit avocado would influence metabolism, whereby the whole avocado would have a favorable effect on morning glycaemia compared with a calorie-equivalent and macronutrient-equivalent evening snack made from different ingredients. The results showed no difference between evening snacks on fasting glucose or peak glucose after a standardized breakfast meal. The area under the glucose curve controlled for baseline [iAUC_(0–80)_] was also not different between the 3 evening snack interventions. Analyses of fasting and postprandial insulin were also not different among interventions. However, the whole fruit avocado snack resulted in lower fasting TG and postbreakfast TG at 180 min. These data suggest the composition of an evening snack may have an important role in TG metabolism.

Snacking behavior contributes to ∼25% of total daily calorie intake, influencing diet quality [1,18]. Snack composition and timing have been researched previously, suggesting that a significant percentage of individuals consume snacks after 18:00 [1]. The idea that previous meal composition can influence metabolic responses to a second meal (e.g. breakfast composition influencing responses to lunch meal) sets the stage for asking questions relative to snacking on the next-meal metabolic responses [19]. The glycemic effects of eating low-GI snacks that emphasize indigestible carbohydrates (i.e. dietary fiber), particularly fermentable fibers producing short-chain fatty acids, the evening before a standardized breakfast meal has been investigated. The data suggested that a healthy evening snack with fibers improves glucose tolerance and lowers inflammatory markers and increases satiety [2,3]. Avocado fruit is a low-GI to no-GI food and contains a reasonable amount of dietary fiber (6.8 g/100 g) in a complex matrix with healthy fats. Previous work has shown replacing carbohydrate calories in a meal with avocado calories improved glucose tolerance in acute single intake feeding paradigms [20,21]. Likewise, avocado intake over 12 wk influenced microbiota composition and microbial-derived metabolite profiles, including short-chain fatty acid (i.e. acetate) generation [22]. Despite these data, we did not detect a difference in next-morning fasting or postbreakfast glycaemia between the avocado and the control snacks. An explanation for the lack of effect may be due to the acute nature of the study. We might have needed several days of avocado snack consumption to observe glycemic benefits, especially if they are dependent on shifts in the metabolic capacity of the gut microbiota. However, Sandberg et al. [3] suggested that 3 days of priming was not necessary when they investigated rye kernels that were processed and made into bread. Avocados are not processed like rye cereal, which influences the fermentability of starch and indigestible starch components. Additionally, our population was characterized by prediabetes, which may require additional priming when unprocessed high-fiber whole foods, such as avocado, are tested for second-meal glycemic benefits.

Although changes in glycemic variables were not evident in this study, we did observe trends for an effect on fasting TG and a significant effect of snack intake on postbreakfast TG concentrations. Leveraging data from the ZOE PREDICT 1 cohort, Bermingham et al. [23] reported snacking behavior on a variety of cardiometabolic outcomes. They found that snacks with lower quality (defined by NOVA) were associated with higher fasting TG and 6-h (AUC) postprandial TG, and higher snack quality was associated with lower fasting and postprandial TG after stratifying on BMI. They also reported that individuals who eat late-evening (after 21:00) and poor-quality snacks had worse fasting and postprandial TG than those who snacked on higher-quality snack foods. The typical snack time for the participants in our study was between 20:00 and 21:00 (2 people consistently ate their snack at 22:00), and the quality of the snacks we tested may be considered high quality (whole avocado) compared with poor quality (control snack: high-carbohydrate, low-fiber, limited micronutrients; matrix control snack: processed ingredient-based product to match macronutrients of whole avocado and limited micronutrients). Thus, the lower TG concentrations in our higher BMI population, particularly postbreakfast, after the whole avocado snack are consistent with these published data.

High carbohydrate–induced hypertriglyceridemia is not a new concept and has been observed in research participants consuming high-carbohydrate, low-fat diets for as few as 5 days [24]. Individuals with higher BMI (≥28) may be more sensitive to lipid and lipoprotein changes when dietary carbohydrate is increased [25]. Generally, high carbohydrate–induced hypertriglyceridemia includes elevated chylomicron and VLDLs. Postprandial elevations are related to fasting TG, and further addition of TG from a meal with fat leads to significantly higher postprandial TG concentrations. The dynamics of TG production from de novo lipogenesis and clearance rate have been investigated to understand the effect of high-carbohydrate diets on the concentration of VLDL particles and VLDL TG [26]. The leading hypothesis for hypertriglyceridemia after high-carbohydrate, low-fat whole food diets in relatively normolipidemic individuals is because of the reduced TG clearance rate [27]. Highly refined simple sugar carbohydrate loads may increase de novo lipogenesis, further contributing to the TG responses [28]. The assembly, production, and clearance of TG after a high carbohydrate diet differs from that of a high-fat diet, highlighting the importance of macronutrient composition on TG metabolism [27]. Noteworthy is that these studies assessing hypertriglyceridemia are in response to days and weeks of consuming a particular dietary composition. This study results were based on 1 snack occasion and a shortened postprandial sampling period focused on glycaemia rather than on the lipidemic responses to different snacks. With the importance of elevated TG as a risk-enhancing factor in cardiovascular diseases and the central role diet and lifestyle modification play in managing hypertriglyceridemia [29], future studies are needed to better understand the effect of snack timing and composition on TG metabolism.

The presented study is novel in testing a whole food (avocado) in an evening snack paradigm on next-morning fasting and postprandial metabolic responses. The research provides intriguing and relevant data to study TG metabolism more deeply in future snack trials with avocado as an evening snack. The project is limited by the duration of sampling to fully characterize the lipid response to the snack compositions, and in retrospect, single-time intake may have been too short to modulate the glycemic effects of the avocado, an unprocessed whole food with healthy fats and fibers. Participants who did not finish the breakfast meal at the clinic reported feeling full and could not finish the meal. We investigated further to determine whether the snack intervention influenced breakfast energy intake or energy density, but no statistical differences in either variable were observed among interventions (*P* > 0.05). Likewise, because participants could choose their own dinner meal to replicate before a visit day, we investigated possible differences in dinner meal intake among snack interventions and according to whether people consumed the entire breakfast or not. There were no statistically significant differences in dinner meals for energy intake (*P* = 0.77), energy density (*P* = 0.63), protein percentage (*P* = 0.97), total fat percentage (*P* = 0.92), carbohydrate percentage (*P* = 0.98), or fiber (*P* = 0.67) among the interventions or by differences in breakfast intake. The reduced sample size for the postprandial analysis was unfortunate due to participants not eating all breakfast meals in their entirety; however, some studies have indicated enhanced satiety effects of certain evening snacks the next morning that may be relevant and open for future investigation.

In summary, consuming an avocado, a high-fiber, high-fat, low-GI food, as an evening snack did not reduce fasting glucose or insulin the next morning or attenuate glucose excursions after the standardized breakfast meal compared with an alternative matrix control snack or high-carbohydrate, low-fiber snack. However, the avocado snack influenced fasting TG and reduced postbreakfast TG at 180 min. These data suggest the composition and unprocessed nature of foods, like the whole food avocado, may have an important role in TG metabolism when consumed as an evening snack by people with prediabetes.

## Author contributions

The authors’ responsibilities were as follows – BMB-F, IE: designed the research; CP, OM, IE, BMB-F: conducted the research; BMB-F, IE: provided essential material; BMB-F: performed statistical analysis; IE, BMB-F, CP: wrote the paper; and all authors: read, revised, and approved the final manuscript.

## Data availability

The data sets generated during and/or analyzed for this study are available from the corresponding author on reasonable request.

## Funding

This study was supported by the Avocado Nutrition Center (ANC).

## Conflict of interest

The authors report no conflicts of interest.
